# Mathematical model of the thalamo-cortical loop by dysfunction in schizophrenia

**DOI:** 10.1186/1471-2202-14-S1-P320

**Published:** 2013-07-08

**Authors:** Nils Rosjat, Silvia Daun-Gruhn, Svitlana Popovych

**Affiliations:** 1 Emmy-Noether Research Group of Computational Biology, Department of Animal Physiology, University of Cologne, 50674 Cologne, Germany

## 

Preceding experimental results suggest that disturbances of auditory information processing within the thalamocortical loop are a core issue relating schizophrenia [1]. Wide differences between schizophrenia patients and healthy controls were found in phase-locking of cortex EEG. We derive a phenomenological mathematical model based on coupled phase oscillators with continuous distributed frequencies to describe the neural activity of the thalamocortical loop. Concerning phase-locking effects observed we examine the influence of the bidirectional coupling strengths between the thalamic and the cortical area. We widen this approach to a model consisting of a thalamic area coupled to three cortical areas, each modeled by a set of nonidentical phase oscillators. At the investigation of our model we use Ott-Antonsen theory [2] and Pikovsky-Rosenblum reduction methods [3]. The results derived from our mathematical model coincide with the experimental data obtained by EEG measurements. The model provides that modifying the coupling strength from the thalamic region to a cortical region effects the duration of phase synchronization and while modifying the coupling back to the thalamic region affects the strength of synchronization in this cortical area. Thus it supports the view that the coupling between the thalamic region and cortical regions is the responsible mechanism for dysfunction of the thalamo-cortical loop in schizophrenia.
